# Hyaluronidase of Bloodsucking Insects and Its Enhancing Effect on *Leishmania* Infection in Mice

**DOI:** 10.1371/journal.pntd.0000294

**Published:** 2008-09-17

**Authors:** Vera Volfova, Jitka Hostomska, Martin Cerny, Jan Votypka, Petr Volf

**Affiliations:** 1 Department of Parasitology, Faculty of Science, Charles University in Prague, Czech Republic; 2 Department of Ecology, Faculty of Science, Charles University in Prague, Czech Republic; National Institute of Allergy and Infectious Diseases, United States of America

## Abstract

**Background:**

Salivary hyaluronidases have been described in a few bloodsucking arthropods. However, very little is known about the presence of this enzyme in various bloodsucking insects and no data are available on its effect on transmitted microorganisms. Here, we studied hyaluronidase activity in thirteen bloodsucking insects belonging to four different orders. In addition, we assessed the effect of hyaluronidase coinoculation on the outcome of *Leishmania major* infection in BALB/c mice.

**Principal Findings:**

High hyaluronidase activity was detected in several Diptera tested, namely deer fly *Chrysops viduatus*, blackflies *Odagmia ornata* and *Eusimilium latipes*, mosquito *Culex quinquefasciatus*, biting midge *Culicoides kibunensis* and sand fly *Phlebotomus papatasi*. Lower activity was detected in cat flea *Ctenocephalides felis*. No activity was found in kissing bug *Rhodnius prolixus*, mosquitoes *Anopheles stephensi* and *Aedes aegypti*, tse-tse fly *Glossina fuscipes*, stable fly *Stomoxys calcitrans* and human louse *Pediculus humanus*. Hyaluronidases of different insects vary substantially in their molecular weight, the structure of the molecule and the sensitivity to reducing conditions or sodium dodecyl sulphate. Hyaluronidase exacerbates skin lesions caused by *Leishmania major*; more severe lesions developed in mice where *L. major* promastigotes were coinjected with hyaluronidase.

**Conclusions:**

High hyaluronidase activities seem to be essential for insects with pool-feeding mode, where they facilitate the enlargement of the feeding lesion and serve as a spreading factor for other pharmacologically active compounds present in saliva. As this enzyme is present in all *Phlebotomus* and *Lutzomyia* species studied to date, it seems to be one of the factors responsible for enhancing activity present in sand fly saliva. We propose that salivary hyaluronidase may facilitate the spread of other vector-borne microorganisms, especially those transmitted by insects with high hyaluronidase activity, namely blackflies (Simuliidae), biting midges (Ceratopogonidae) and horse flies (Tabanidae).

## Author Summary

Hyaluronidases are enzymes degrading the extracellular matrix of vertebrates. Bloodsucking insects use them to cleave the skin of the host, enlarge the feeding lesion and acquire the blood meal. In addition, resulting fragments of extracellular matrix modulate local immune response of the host, which may positively affect transmission of vector-borne diseases, including leishmaniasis. Leishmaniases are diseases with a wide spectrum of clinical forms, from a relatively mild cutaneous affection to life-threatening visceral disease. Their causative agents, protozoans of the genus *Leishmania*, are transmitted by phlebotomine sand flies. Sand fly saliva was described to enhance *Leishmania* infection, but the information about molecules responsible for this exacerbating effect is still very limited. In the present work we demonstrated hyaluronidase activity in salivary glands of various Diptera and in fleas. In addition, we showed that hyaluronidase exacerbates *Leishmania* lesions in mice and propose that salivary hyaluronidase may facilitate the spread of other vector-borne microorganisms.

## Introduction

Hyaluronidases are a family of enzymes that degrade hyaluronan (HA) and several other glycosaminoglycan constituents of the extracellular matrix of vertebrates (for review see [1]). In insects, hyaluronidases are well-known from venoms of Hymenoptera and represent clinically important allergens of honey-bees, wasps and hornets [2]–[4]. Hyaluronidases were found also in cDNA libraries of salivary glands (sialomes) of various bloodsucking insects [5]–[8] and the enzyme activity was found in saliva of three groups of Diptera, namely sand flies, blackflies, and horse flies [9],[10]. Salivary hyaluronidases of parasitic insects may have diverse effects on the host. They play an important role in blood meal acquisition; by degrading HA abundant in host skin, hyaluronidases increase tissue permeability for other salivary components that serve as antihaemostatic, vasodilatory or anti-inflammatory agents [5],[9]. This is why hyaluronidases are frequently called “spreading factors” [11]. The enzyme activity facilitates the enlargement of the feeding lesion and the insect acquires the blood meal more rapidly. In addition, HA fragments were shown to have immunomodulatory properties; they affect maturation and migration of dendritic cells, induction of iNOS and chemokine secretion by macrophages and proliferation of activated T cells (reviewed in [12]). As blood sucking insects represent the most important vectors of infectious diseases, local immunomodulation of the vertebrate host may positively enhance the infection.

Leishmaniasis is one of the most prevalent vector-borne diseases. It is initiated by the intradermal inoculation of *Leishmania* promastigotes during the bite of an infected sand fly (Diptera: Phlebotominae). As shown first by Titus and Ribeiro [13] saliva of the sand fly vector exacerbates the initial phase of *Leishmania* infections in terms of parasite burden and size of the cutaneous lesion. Sand fly saliva was described to contain an array of pharmacologically active compounds affecting host hemostasis and immune mechanisms (reviewed in [14],[15]) but the information about molecules responsible for the exacerbating effect is still very limited. Morris et al. [16] showed that maxadilan, a well-known vasodilator of the New World vector *Lutzomyia longipalpis*, exacerbates *Leishmania* infection to the same degree as whole saliva. Maxadilan inhibits splenocyte proliferation induced *in vitro* and delayed type hypersensitivity in mice [17] and it also has several inhibitory effects on macrophages and monocytes that would support *Leishmania* survival in the host [18]. However, this important peptide was not found in Old World vectors of genus *Phlebotomus* (www.ncbi.nih.gov), including *P. papatasi* where exacerbating effect of saliva was repeatedly demonstrated [19],[20]. The vasodilatory activity of *P. papatasi* was instead ascribed to adenosine and AMP present in saliva of this sand fly [21].

In the present work, we studied hyaluronidase activity in bloodsucking insects of four different orders. In addition, we assessed the effect of hyaluronidase coinoculation on the outcome of *Leishmania major* skin lesions and spreading into draining lymph nodes.

## Materials and Methods

### Insects and preparation of samples

Samples used are summarized in Table 1. The insects originated from laboratory colonies or were collected in the wild. Salivary glands were dissected out in Tris buffer (20 mM Tris, 150 mM NaCl, pH 7.8) and stored in batches (usually 20 glands in 20 µl of Tris buffer) at −70°C. Where dissection of salivary glands was not feasible, whole bodies (*Ctenocephalides* flea, *Culicoides* midge) or the thoracic parts containing salivary glands (*Pediculus* louse) were used at protein concentration 20 µg/µl. Salivary gland extracts (SGE) or body extracts (BE) were obtained by disruption of tissue by three freeze-thaw cycles in liquid nitrogen, homogenization and centrifugation at 12,000 g for 5 min. Protein concentration was determined by Bradford assay using bovine serum albumin in Tris buffer as a standard.

**Table 1:** Origin of insects used and the estimated protein content per one salivary gland.

Sample	Origin	µg protein per salivary gland
*Aedes aegypti* SGE	colony, Inst. Parasitol., Ceske Budejovice, CZ	0.67
*Anopheles stephensi* SGE	colony, University of Aberdeen, UK	0.41
*Ctenocephalides felis* BE	colony, Bayer CropScience, Germany	nd.
*Culicoides kibunensis* BE	field, Prague, CZ	nd.
*Culicoides pictipennis* BE	field, Prague, CZ	nd.
*Culex quinquefasciatus* SGE	colony, Dept. Parasitol., Charles University, CZ	0.39
*Eusimulium latipes* SGE	field, Prague, CZ	0.83
*Glossina fuscipes* SGE	colony, CSIRA, Montpellier, Francie	7
*Chrysops viduatus* SGE	field, Veseli, CZ	6.9
*Odagmia ornata* SGE	field, Veseli, CZ	nd.
*Pediculus humanus* TE	colony, Bayer CropScience, Germany	nd.
*Phlebotomus papatasi* SGE	colony, Dept. Parasitol., Charles University, CZ	0,4
*Rhodnius prolixus* SGE	colony, Inst. Parasitol., Ceske Budejovice, CZ	20.4
*Stomoxys calcitrans* SGE	colony, University of Wales, UK	1.2

SGE – salivary gland extract, BE – body extract, TE – thoracic extract, nd. – not determined.

### Detection of hyaluronidase activity

Enzyme activity was detected by the dot method on 10% polyacrylamide gels with copolymerized hyaluronic acid (HA, potassium salt, from human umbilical cord, ICN Pharmaceutical, CA). Gels were prepared using 0.1 M acetate, pH 5.5, containing 0.1 M NaCl, 0.05% Tween-20 and 0.002% HA. This method was previously proved as sensitive and reproducible [10]. Preliminary experiment with selected salivary extracts revealed that *Phlebotomus papatasi* and *Culex pipiens* samples were positive at pH 4.5, 5.5, 6.5 and 7.5 while *Aedes aegypti*, *Anopheles stephensi* and *Glossina fuscipes* samples were consistently negative (Fig. S1). Therefore pH 5.5 was chosen for this assay as this is about the pH optimum known for salivary hyaluronidases of various Diptera [9],[10]. Insect samples (2 µl volume) were dotted on the gel and sheep testicular hyaluronidase, (Sigma, 1 µg in 1 µl) was used as a control. Incubation was carried out for 24 hrs at 37°C in a moist chamber. The gels were then washed in water, soaked in 50% formamide for 30 min and stained in Stains-all (Sigma) solution (100 µg/ml in 50% formamide) for 24 hrs in the dark. After a rinse in distilled water the gels were scanned and photographed. To determine whether the enzyme activity was specific for cleaving HA, we tested positive samples also with another component of extracellular matrix, chondroitin sulfate. The method was performed as described above, only HA was replaced by 0.002% chondroitin sulfate (Sigma).

### Electrophoretic analysis of hyaluronidase activity

Electrophoresis (SDS PAGE) was carried out on 10% slab gels (0.75 mm thick) using Mini-Protean II apparatus (Biorad) and constant voltage 150 V. Substrate gels were copolymerized with 0.002% HA. As the hyaluronidase activities and band patterns varied among insects, different loads were used per lane in order to obtain bands of equal intensity. Following electrophoresis, gels were rinsed 2×20 min in 0.1 M Tris, pH 7.8, 20 min in 0.1 M acetate buffer, pH 5.5 (both with 1% Triton X-100 to wash out SDS) and then incubated in 0.1 M acetate buffer (without detergent) for 120 min at 37°C. After rinsing in water the gels were stained with Stains-all as described above. Hyaluronidase activity was visible as a pink band on a dark blue background.

### Hyaluronidase effect on *Leishmania* infection in mice

Experiments on mice were done in accordance with Czech Act No. 246/1992 and approved by IACUC of the Fac. Sci., Charles University in Prague. A mouse ear infection model [19] was used to assess the effect of hyaluronidase coinoculation on the outcome of *Leishmania* infection. *Leishmania major* clone LV561 (MHOM/IL/67/LRC-L137 Jericho II) was cultured on blood agar from defibrinated rabbit blood, supplemented with 50 µg/ml gentamicin. Female BALB/c mice (Charles River Deutschland, Sulzfeld, Germany) were used at the age of 8 weeks. Ether-anaesthetized mice were inoculated in the ear dermis with 10^4^ or 10^5^ *L. major* stationary-phase promastigotes (subculture 1) in 5 µl sterile saline. The inoculum also contained bovine testicular hyaluronidase (Sigma) in an amount equivalent to 2 or 10 “optimal salivary glands” of *Phlebotomus papatasi* [10], i.e. 0.4 and 2.0 relative turbidity reducing units, respectively. Bovine testicular hyaluronidase belongs to the same enzyme class as the sand fly salivary hyaluronidases [10] and shares sequence homology with the enzyme of *L. longipalpis* [5]. Control animals were inoculated with parasites in sterile saline only. Sixty mice (10 for each of six groups) were used for Q-PCR and another 48 (8 for each group) for lesion monitoring. The size of skin lesions was measured weekly using a Vernier caliper gauge. Lesions were monitored for 6 weeks post infection: the area was calculated from two perpendicular measurements as an ellipse area, and its appearance (degree of ulceration) was assessed using an arbitrary scale from 1 to 5 (1 - low induration, 2 - high induration, 3 - small ulcer, 4 - large ulcer, 5 - perforated ear pinna). Independently in both parasite doses (10^4^ and 10^5^), the significance of the hyaluronidase effect was tested using nonparametric Kruskal-Wallis ANOVA and post hoc comparisons of mean ranks using Statistica 7 routines [22]. The tests were performed separately for weeks 3, 4, 5, and 6 post-infection; the size of a lesion was calculated as its area weighted by the degree of ulceration.

### Detection and quantification of *Leishmania* parasites in mice

Mice were sacrificed 24 hrs post inoculation (p.i.) as the preliminary experiment revealed that lymph nodes of mice dissected 24 hours p.i. gave more consistent results than those dissected 48 hours p.i. (Fig. S2). Parasite numbers in draining retromaxillar lymph nodes were determined by quantitative PCR (Q-PCR) as described earlier [23]. Briefly, dissected lymph nodes were stored in 10 µl saline at −70°C. Total DNA was isolated from homogenised samples using High Pure PCR Template Preparation Kit (Roche); kinetoplast DNA was targeted using primers described elsewhere [24]. The relative effectiveness of three hyaluronidase doses (equivalent to 0, 2, and 10 *P. papatasi* salivary glands) with both infection doses (10^4^ and 10^5^ parasites) was evaluated by analysis of variance (Statistica v. 7.1, factorial and one-way ANOVA).

## Results

### Detection of hyaluronidase activity

**Figure 1:**
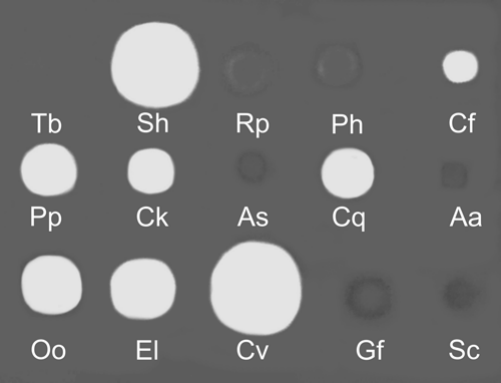
Hyaluronidase activity of insects tested by dot method on polyacrylamide gel with incorporated hyaluronan. Protein content per 2 µl dot is indicated in brackets. Tb = Tris buffer, Sh = sheep testicular hyaluronidase (10 µg), Rp = *Rhodnius prolixus* SGE (20 µg), Ph = *Pediculus humanus* BE (20 µg), Cf = *Ctenocephalides felis* BE (20 µg), Pp = *Phlebotomus papatasi* SGE (0.8 µg), Ck = *Culicoides kibunensis* BE (20 µg), As = *Anopheles stephensi* SGE (0.8 µg), Cq = *Culex quinquefasciatus* SGE (0.8 µg), Aa = *Aedes aegypti* SGE (1.3 µg), Oo = *Odagmia ornata* SGE (0.8 µg), El = *Eusimulium latipes* SGE (1.7 µg), Cv = *Chrysops viduatus* SGE (3.5 µg), Gf = *Glossina fuscipes* SGE (14 µg), Sc = *Stomoxys calcitrans* SGE (2.4 µg).

**Figure 2:**
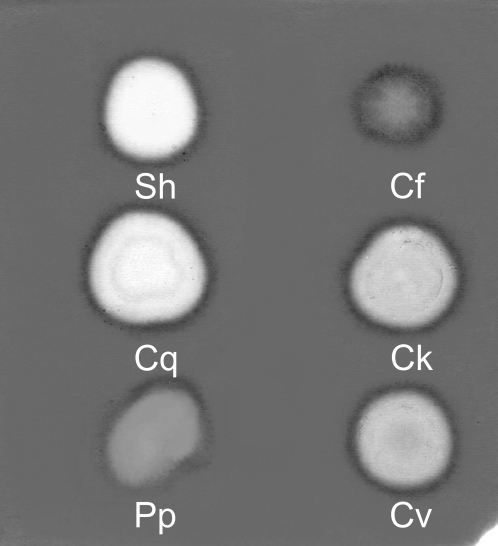
Substrate specificity of hyaluronidases tested on polyacrylamide gel with incorporated chondroitin sulfate. Protein content per 2 µl dot is indicated in brackets. Sh = sheep testicular hyaluronidase (10 µg), Cf = *Ctenocephalides felis* SGE (20 µg), Cq = *Culex quinquefasciatus* SGE (0.8 µg), Ck = *Culicoides kibunensis* BE (20 µg), Pp = *Phlebotomus papatasi* SGE (0.8 µg), Cv = *Chrysops viduatus* SGE (0.8 µg).

The dot method on gels with copolymerized HA and chondroitin sulfate was used to study the presence of hyaluronidase activity and its substrate specificity. The highest hydrolysis of HA was observed in SGE of deer fly *Chrysops viduatus*. Pronounced hydrolysis was found in SGEs of blackflies *Odagmia ornata* and *Eusimulium latipes*, mosquito *Culex quinquefasciatus*, sand fly *Phlebotomus papatasi* and whole body extract of biting midge *Culicoides kibunensis* (syn. *C. cubitalis*). Lower activity was detected in BE of cat flea *Ctenocephalides felis* (Fig. 1). On the other hand, no detectable hydrolysis of HA occurred in SGEs of kissing bug *Rhodnius prolixus*, mosquitoes *Anopheles stephensi* and *Aedes aegypti*, tse-tse fly *Glossina fuscipes*, stable fly *Stomoxys calcitrans* and in thoracic extracts of human louse *Pediculus humanus* (Fig. 1). Positive samples were then tested also for chondroitin sulfate hydrolysis (Fig. 2). High activity was observed in *Culex quinquefasciatus* and *Culicoides kibunensis*, in other samples the hydrolysis of chondroitin sulfate was either moderate (*Chrysops viduatus*) or low (*Phlebotomus papatasi*, *Ctenocephalides felis*) (Fig. 2); clearly, HA is the preferred substrate for the enzymes of these three insects.

### Electrophoretic analysis of hyaluronidase activity

**Figure 3:**
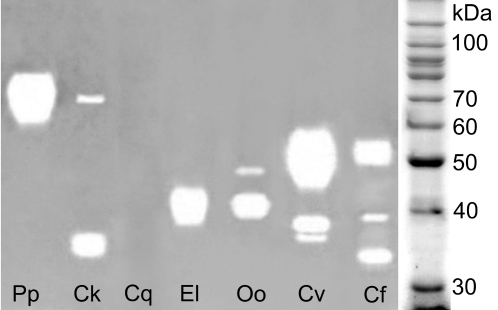
SDS PAGE zymography under nonreducing conditions on polyacrylamide gel with incorporated hyaluronan. Protein content per lane is given in brackets. Pp = *Phlebotomus papatasi* SGE (0.2 µg), Ck = *Culicoides kibunensis* BE (10 µg), Cq = *Culex quinquefasciatus* SGE (5.6 µg), El = *Eusimulium latipes* SGE (0.4 µg), Oo = *Odagmia ornata* SGE (0.4 µg), Cv = *Chrysops viduatus* SGE (0.2 µg), Cf = *Ctenocephalides felis* BE (15 µg).

**Figure 4:**
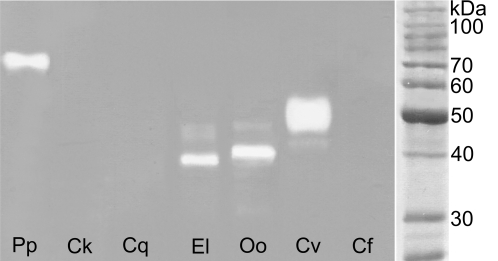
SDS PAGE zymography on the same gel as in Figure 3 but under reducing conditions. Pp = *Phlebotomus papatasi* SGE (0.2 µg), Ck = *Culicoides kibunensis* BE (20 µg), Cq = *Culex quinquefasciatus* SGE (8 µg), El = *Eusimulium latipes* SGE (3 µg), Oo = *Odagmia ornata* SGE (3 µg), Cv = *Chrysops viduatus* SGE (0.3 µg), Cf = *Ctenocephalides felis* BE (15 µg).

Seven samples positive in the dot method were analyzed by zymography to reveal the apparent molecular weight (MW) of hyaluronidases. The MW of the enzymes differed among various insects (Figs. 3 and 4). Under nonreducing conditions hyaluronidases were detected as major diffuse bands (Fig. 3). The SGE activity in *Phlebotomus papatasi* had a MW about 70 kDa while those in both blackfly species tested, *Eusimulium latipes*, and *Odagmia ornata*, about 40 kDa. In BE of *Culicoides kibunensis*, the major band of about 35 kDa was accompanied with a minor one of 70 kDa, supposedly a dimer. *Chrysops viduatus* SGE revealed one major band with estimated MW of 50 kDa. In BE of flea *Ctenocephalides felis*, three enzyme bands were detected, the most prominent one of about 52 kDa (Fig. 3). Under reducing conditions, SDS PAGE revealed sharper enzyme bands allowing more precise assignment of corresponding MW (Fig. 4). In sand fly *P. papatasi*, both blackfly species and deer fly *Chrysops viduatus*, hyaluronidase activity was observed within the same MW ranges as under nonreducing conditions (70, 40 kDa, and 50 kDa, respectively). In *Culicoides kibunensis* and *Ctenocephalides felis* hyaluronidase activity was not detectable under reducing conditions (Fig. 4).

**Figure 5:**
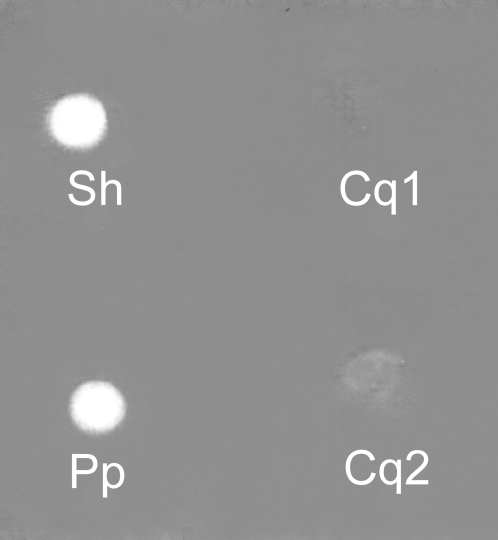
Effect of SDS on hyaluronidase activity in SGE of *Culex quinquefasciatus*. Activity of hyaluronidase was tested by dot method on polyacrylamide gel with incorporated 0.002% hyaluronan and 0.001% SDS. Protein content per 2 µl dot is indicated in brackets. Sh = sheep testicular hyaluronidase (2 µg), Pp = *Phlebotomus papatasi* SGE (0.8 µg), Cq1 = *Culex quinquefasciatus* SGE (1.6 µg), Cq2 = *Culex quinquefasciatus* SGE (3.2 µg).

No hyaluronidase activity was detected in *Culex quinquefasciatus* SGE under either zymography conditions used, reducing and nonreducing. An additional experiment was performed to explain the contradictory results from the dot method and zymography; SGE of *C. quinquefasciatus* was dotted on the gel with copolymerized HA with and without the presence of SDS. Hydrolysis was observed only in the sample without SDS (Fig. 5).

### Hyaluronidase effect on *Leishmania* infection in mice

**Figure 6:**
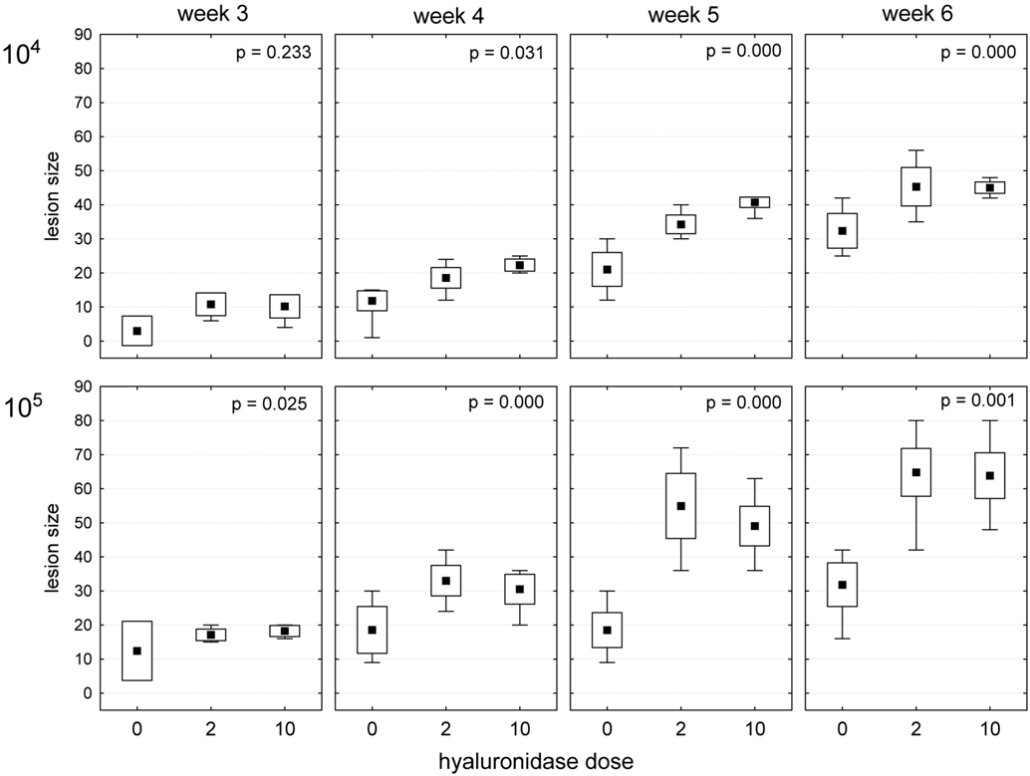
Effect of hyaluronidase on *Leishmania* infection in mice. BALB/c mice were coinoculated intradermally into ear with 10^4^ or 10^5^ *Leishmania major* and hyaluronidase equivalent to 0, 2 and 10 salivary glands of *Phlebotomus papatasi*. Lesion size, given as a product of its area (mm^2^) and the degree of ulceration (1–5), was monitored for 6 weeks post infection. Points (▪) = mean values, boxes = 95% confidence intervals, whiskers = min-max values. The *p* values of corresponding Kruskal-Wallis ANOVA are provided.

Next we examined whether hyaluronidase altered the course of *Leishmania major* infection in BALB/c mice. We used intradermal inoculation into the ear and the disease burden was calculated from weekly measuring the lesion size. As shown in Fig. 6, mice coinjected with parasites and hyaluronidase developed bigger lesions. In all groups of mice, the onset of lesion development was at three weeks p.i. Thereafter, the lesions grew faster in coinoculated groups. The experiment was terminated six weeks post infection when, in some animals, ulcerating lesion spread over the majority of ear pinna. In mice inoculated by higher parasite numbers (10^5^), both hyaluronidase treatments produced similar effects (Fig. 6A). In mice with an inoculation dose one order of magnitude lower (10^4^), the effect of hyaluronidase was concentration-dependent: lesions were bigger in mice coinoculated with hyaluronidase activity equivalent of 10 *P. papatasi* salivary glands than in those coinoculated with equivalent of 2 glands (Fig. 6B). In both parasite numbers (10^4^ and 10^5^) over all considered weeks (3 to 6) post-inoculation, Kruskal- Wallis ANOVA showed significant differences among hyaluronidase treatments (*p* always≤0.025), with only one exception in week 3 of 10^4^ parasites treatment (*p* = 0.23). Consequently, the post-hoc comparison of treatments tests confirmed the significant difference between controls (no hyaluronidase) and corresponding inoculated hyaluronidase doses (2 or 10 glands equivalents). We also tested the difference between the 2 and 10 gland equivalent doses: however, despite the common trends apparent in Fig. 6 indicating that there may be a systematic difference between 2 and 10 gland equivalents doses, the post-hoc comparison of treatments test did not prove it in any case but in week 5 of the 10^4^ parasites treatment.

We also examined whether hyaluronidase affected *Leishmania major* load in draining lymph nodes of BALB/c mice one day p.i. Using Q-PCR, no significant differences were observed among control and experimental groups of mice at both parasite doses (10^4^ or 10^5^ *L. major*) tested (F_(2, 54)_ = 0.043; p = 0.96) (Fig. S3).

## Discussion

Parasitic insects utilize two strategies for finding blood: solenophagy (or vessel feeding) and telmophagy (or pool feeding). In solenophagic approach, the feeding fascicle cannulates a blood vessel, while in the pool-feeding mode the mouth part stylets slash through the skin, and the insect sips blood that oozes out from the hemorrhage. In our experiments, pronounced hyaluronidase activity was found in black flies, biting midges, sand flies and deer flies. All these insects belong to parasitic Diptera with pool-feeding mode of blood meal acquisition. The activity was detected also in cat flea (*Ctenocephalides felis*, Siphonaptera) and in *Culex quinquefasciatus* mosquito (Diptera). Although these two species belong to different insect orders, they are both vessel feeders. In contrast, no activity was detected in other vessel-feeding insects: human lice, kissing bugs, *Anopheles* and *Aedes* mosquitoes, tsetse flies, and stable flies.

Hyaluronidase activity was previously detected in the saliva of various sand fly species [9],[10] as well as in the saliva of the black fly *Simulium vittatum* [9] and horse fly *Tabanus yao* [8]. Sequences predicted to code for hyaluronidases were found in the salivary transcriptomes of the mosquito *Culex quinquefasciatus* [6] and the biting midge *Culicoides sonorensis* [7]. Herein, we demonstrated that *Culex quinquefasciatus* and *Culicoides kibunensis* possess hyaluronidase activity and, in parallel experiments, we detected hyaluronidase activity in saliva of two other species of biting midges *Culicoides sonorensis* and *C. nubeculosus* (Volfova et al., unpublished). Therefore, we showed that in biting midges and in *Culex quinquefasciatus*, the transcripts coding for putative hyaluronidases are translated into functional enzymes.

To determine whether the enzyme activity was specific for cleaving HA, we also tested another component of mammalian extracellular matrix, chondroitin sulfate. All hyaluronidase-positive samples tested cleaved chondroitin sulfate, which would indicate that insect hyaluronidases fall into the same class as mammalian hyaluronidases (E.C. 3.2.1.35 according to IUBMB Enzyme Nomenclature) [25]. Indeed, sequence analysis of transcripts putatively coding for hyaluronidase enzymes reveals their homology to mammalian enzymes [6],[7],[9]. While HA was found as the preferred substrate for most samples tested, very high hydrolysis of chondroitin sulfate was found in *Culex quinquefasciatus* SGE. This mosquito species differs from other samples tested also in other aspects. In zymography assay, salivary hyaluronidase of *Culex quinquefasciatus* was irreversibly sensitive to denaturation effect of SDS while enzymes of other insects tested refolded and regained activity after removal of the denaturating agent. Further work is needed to understand the differences in the molecular structure and substrate specificity of hyaluronidases from *Culex* mosquitoes versus other bloodsucking insects. In addition, in other mosquitoes studied, *Anopheles darlingi* [26], *funestus* [27] and *gambiae* [28] and *Aedes aegypti* [29] and *albopictus* [30], neither hyaluronidase activity nor hyaluronidase gene was found in salivary transcriptomes. Sequences homologous to hyaluronidase were, however, found by genome sequencing in *Anopheles gambiae* [31] and *Aedes aegypti* [32].

As revealed by zymography, hyaluronidases of different insect species tested vary substantially in MW and the structure of the molecule. Putative oligomers were seen in *Culicoides kibunensis*. Oligomeric forms have been found frequently among mammalian hyaluronidases. In sand flies, oligomers or dimers were found in *Lutzomyia longipalpis*, *Phlebotomus papatasi*, and *P. sergenti* [10]. Multiple bands observed by zymography in *Ctenocephalides felis* whole body extract could, however, represent multiple hyaluronidase enzymes.

In *Culicoides* and *Ctenocephalides*, reducing conditions affected the stability of the enzymes; 2-mercaptoethanol inhibited hyaluronidase activity. In *Culicoides* midges, the sensitivity of hyaluronidase activity to reducing conditions was confirmed by experiments with pure saliva of laboratory bred *Culicoides sonorensis* and *C. nubeculosus* (Volfova et al., unpublished). This implies that reduction-sensitive residues are either important for the function of the active site of the enzyme, or steric relations in the molecule. On the other hand, hyaluronidases of other insects tested, namely *Phlebotomus papatasi*, *Eusimulium latipes*, *Odagmia ornata*, and *Chrysops viduatus*, remained active under reducing conditions. Addition of 2-mercaptoethanol did not result in differences in the apparent MW, suggesting that the enzymes consist of a single polypeptide chain. These results correspond with previous observations on sand flies; sand fly hyaluronidases strikingly differed in structure and sensitivity to reducing conditions, even among various species of the genus *Phlebotomus* [10].

We showed that hyaluronidase is a common constituent of saliva of bloodsucking insects. It seems to be essential for insects with pool-feeding mode, where it facilitates the enlargement of the feeding lesion, serving as a spreading factor for other pharmacologically active compounds present in saliva. Very little is known, however, about the possible role of salivary gland hyaluronidase in allergic reactions which occur in some patients after repeated bites of bloodsucking Diptera. In Hymenoptera, venom hyaluronidase is largely responsible for the cross-reactivity of venoms with sera of allergic patients [4]. In several patients, coexistent anaphylaxis to Hymenoptera sting and Diptera bite was described [33] and hyaluronidase is a candidate allergen responsible for this type of crossreactions. In experiments of Sabbah et al. [34],[35], IgE of allergic patients recognized shared proteins within MW range 44–50 kDa between wasp venom and total extracts of mosquito and horse fly. Unfortunately, these interesting data are difficult to assess given the incomplete identitification of the mosquito and horse fly species tested.

A mouse ear infection model was used to assess the effect of hyaluronidase coinoculation on the outcome of *Leishmania major* infection. The activity of sand fly enzyme was mimicked by commercially available bovine hyaluronidase. More severe lesions developed in mice where *L. major* promastigotes were coinjected with hyaluronidase. Even the lower dose of the enzyme corresponding with the activity produced by 1–2 sand fly females resulted in significant differences against the control mice where parasites alone were injected. It would be worth testing if differences observed in lesion size are mainly due to number of parasites or to inflammatory response to coinoculated hyaluronidase. In contrast, there was neither more rapid onset of lesions, nor faster dissemination of *Leishmania* in the lymph node. Parasite numbers in draining lymph nodes collected 24 and 48 hrs p.i. were similar in all experimental groups. Although hyaluronidase activity exacerbated *Leishmania* lesions in the skin, it did not support its visceralization. However, we can not exclude the possibility that consequences of hyaluronidase for parasite visceralization are not immediate and thus could not be detected in the present study.

The way by which hyaluronidase enhances the establishment of *Leishmania* is unknown, but we suggest that it is due to HA fragments generated by hyaluronidase activity in the host skin. HA occurs in two main forms: the high MW (HMW) polymers and the low MW (LMW) fragments. HMW HA is a common component of vertebrate extracellular matrix. LMW HA fragments are generated under inflammatory conditions by endogenous or bacterial hyaluronidases [36], or non-enzymatically by free radicals [37]. HA fragments have diverse immunomodulatory properties; they affect DC maturation, T cell proliferation, cytokine, and chemokine synthesis by lymphocytes and macrophages (reviewed in [12]). Thus, following injury or infection, HA fragments have been implicated as both endogenous and exogenous triggers of repair and/or defense mechanisms [38],[39] and might truly represent a “danger signal” [40]. *Leishmania* parasites, however, may profit from the local increase of HA fragments. Specifically, endothelial cells were shown to respond to LMW HA by IL-8 production [38] that results in neutrophil recruitment. As neutrophil granulocytes were indicated as Trojan horses enabling *Leishmania* silent entry into macrophages [41] their accumulation at the site of sand fly bite might promote infection establishment.

In conclusion, we demonstrated that hyaluronidase promotes *Leishmania* establishment in murine skin. As this enzyme is present in all *Phlebotomus* and *Lutzomyia* species studied to date [10] it seems to be one of the factors responsible for enhancing activity present in saliva of the New-World as well as the Old-World sand flies. We propose that hyaluronidase, in concert with other insect-derived molecules, may facilitate the spread of other vector-borne diseases, especially those transmitted by vectors with high hyaluronidase activity in saliva, namely blackflies, biting midges, deer flies and horse flies.

## Supporting Information

Figure S1 Effect of pH on hyaluronidase activity in SGE of various bloodsucking insects. Activity of hyaluronidase was tested by dot method on polyacrylamide gel with incorporated 0.002% hyaluronan. Four different pH were compared: pH 4.5, pH 5.5 (both 0.1 M acetate buffer), pH 6.5 (0.1 M bis-Tris buffer) and pH 7.5 (0.1 M Tris buffer). Protein content per 2 µl dot is indicated in brackets. Sh = sheep testicular hyaluronidase (2 µg), Pp = *Phlebotomus papatasi* SGE (0.8 µg), As = *Anopheles stephensi* SGE (0.8 µg), Cq = *Culex quinquefasciatus* SGE (0.8 µg), Aa = *Aedes aegypti* SGE (1.3 µg), Gf = *Glossina fuscipes SGE* (14 µg). Samples were incubated for 24 hours at 37°C.
(0.60 MB TIF)

Figure S2 Effect of hyaluronidase on Leishmania major numbers in draining lymph nodes 48 hrs post infection. BALB/c mice were coinoculated intradermally into ear with 104 (A) or 105 (B) *Leishmania major* and hyaluronidase equivalent to 0, 2 and 10 salivary glands of *Phlebotomus papatasi*. Points (▪) = mean values; vertical bars = 95% confidence intervals.
(0.46 MB TIF)

Figure S3 Effect of hyaluronidase on Leishmania major numbers in draining lymph nodes 24 hrs post inoculation. BALB/c mice were coinoculated intradermally into ear with 104 (A) or 105 (B) *Leishmania major* and hyaluronidase equivalent to 0, 2 and 10 salivary glands of *Phlebotomus papatasi*. Points (▪) = mean values; vertical bars = 95% confidence intervals; A: 104 parasites, one-way ANOVA F(2, 27) = 1.989, p = 0.16; B: 105 parasites, one-way ANOVA F(2, 27) = 0.145, p = 0.87; C: 104 and 105 parasites combined, factorial ANOVA F(2, 54) = 0.043, p = 0.96.
(0.38 MB TIF)
