# Supplementary figures and images for: Hyaluronidase of Bloodsucking Insects and Its Enhancing Effect on Leishmania Infection in Mice
Source: PLoS Negl Trop Dis. 2008 Sep 17;2(9):e294. doi: 10.1371/journal.pntd.0000294 (PMC2553483; doi:10.1371/journal.pntd.0000294)

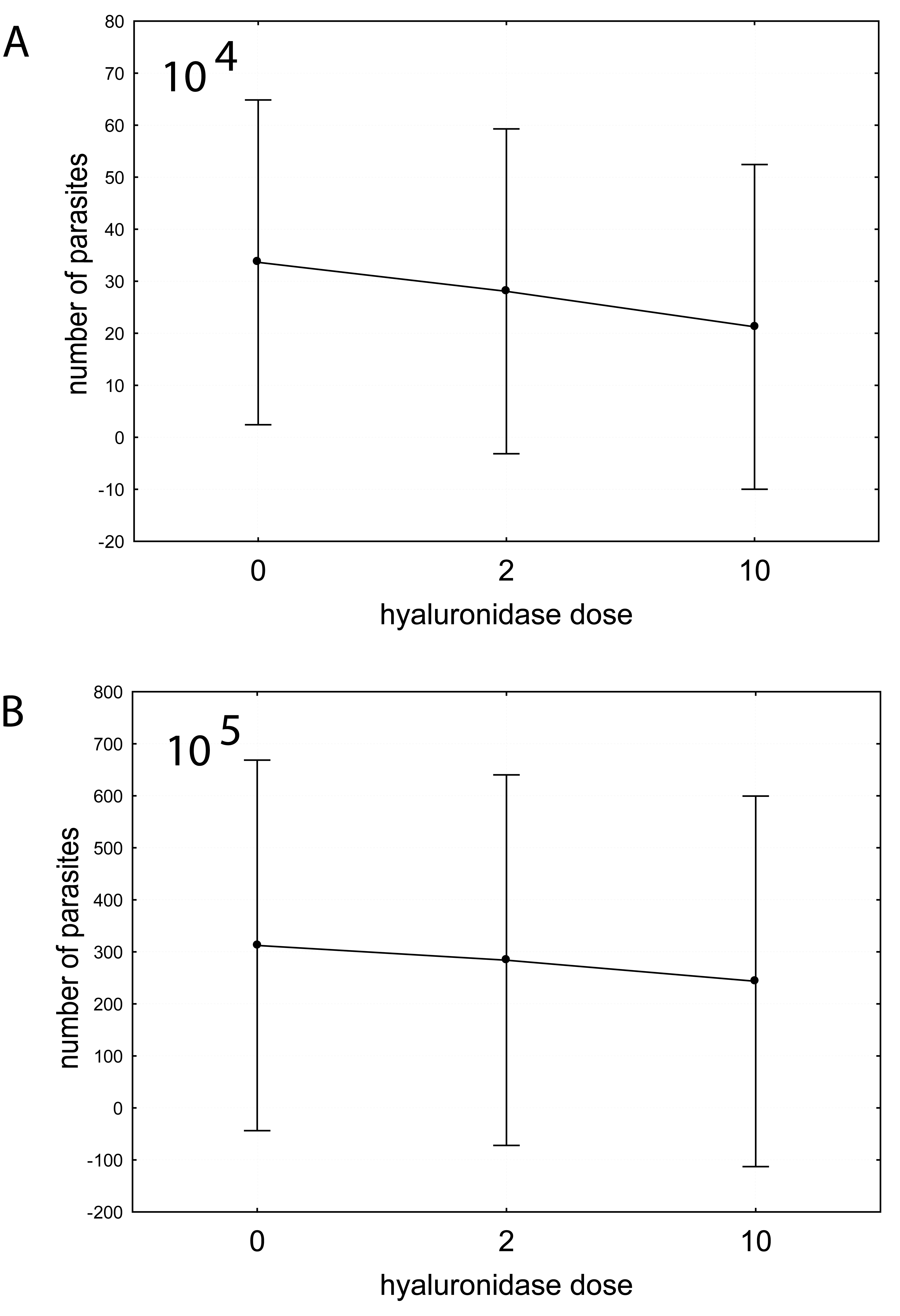

Supplement: Figure S2 — Effect of hyaluronidase on Leishmania major numbers in draining lymph nodes 48 hrs post infection. BALB/c mice were coinoculated intradermally into ear with 104 (A) or 105 (B) Leishmania major and hyaluronidase equivalent to 0, 2 and 10 salivary glands of Phlebotomus papatasi. Points (▪) = mean values; vertical bars = 95% confidence intervals. (0.46 MB TIF) [file pntd.0000294.s002.tif]

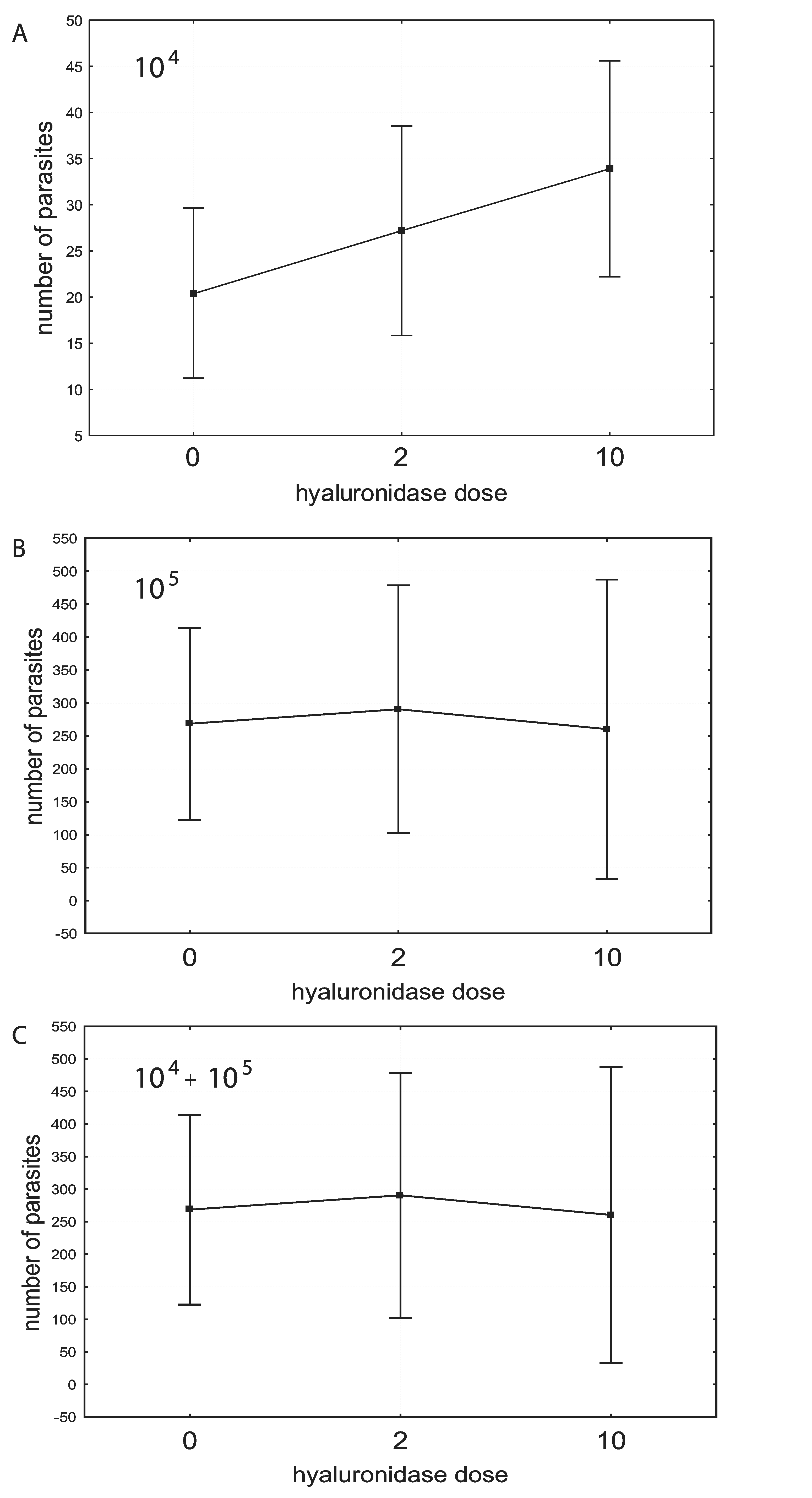

Supplement: Figure S3 — Effect of hyaluronidase on Leishmania major numbers in draining lymph nodes 24 hrs post inoculation. BALB/c mice were coinoculated intradermally into ear with 104 (A) or 105 (B) Leishmania major and hyaluronidase equivalent to 0, 2 and 10 salivary glands of Phlebotomus papatasi. Points (▪) = mean values; vertical bars = 95% confidence intervals; A: 104 parasites, one-way ANOVA F(2, 27) = 1.989, p = 0.16; B: 105 parasites, one-way ANOVA F(2, 27) = 0.145, p = 0.87; C: 104 and 105 parasites combined, factorial ANOVA F(2, 54) = 0.043, p = 0.96. (0.38 MB TIF) [file pntd.0000294.s003.tif]
